# In Vitro Activity of Manuka Honey, Either Alone or in Combination With Topical Antibiotics, Against Bacteria Commonly Found in Equine Ulcerative Keratitis

**DOI:** 10.1111/vop.70111

**Published:** 2025-11-12

**Authors:** M. Barvelink, B. Brok, S. C. Djajadiningrat‐Laanen, J. C. M. Vernooij, E. M. Broens, I. J. M. Slenter

**Affiliations:** ^1^ Department of Clinical Sciences, Equine Sciences, Faculty of Veterinary Medicine Utrecht University Utrecht the Netherlands; ^2^ Department of Clinical Sciences, Surgery of Companion Animals, Veterinary Ophthalmology Section, Faculty of Veterinary Medicine Utrecht University Utrecht the Netherlands; ^3^ Department of Population Health Sciences, Farm Animal Health, Faculty of Veterinary Medicine Utrecht University Utrecht the Netherlands; ^4^ Department of Biomolecular Health Sciences, Veterinary Microbiological Diagnostic Centre, Faculty of Veterinary Medicine Utrecht University Utrecht the Netherlands

**Keywords:** antibiotic resistance, cornea, eye, horse, methicillin‐resistant, multidrug resistance

## Abstract

**Objective:**

To assess the antibacterial activity of manuka honey against bacterial isolates commonly associated with infected corneal ulcerations in horses, and to investigate possible combined effects of manuka honey and commonly prescribed topical antibiotics.

**Procedures:**

Four *Staphylococcus aureus* , including three methicillin‐resistant (MRSA), two methicillin‐resistant coagulase‐negative staphylococci ( *S. sciuri* and *S. haemolyticus* ), and two *Streptococcus equi* subspecies *zooepidemicus* isolates from horses with stromal ulcerative keratitis were selected. Minimum inhibitory concentrations (MICs) and minimum bactericidal concentrations (MBCs) of manuka honey were determined with microdilution assays and spectrophotometric analyses. Potential combined antibacterial effects were explored by assessing bacterial growth inhibition using disk diffusion and E‐tests, both with and without a sub‐inhibitory concentration of manuka honey. Tested antibiotics included chloramphenicol, tetracycline, gentamicin, ofloxacin, tobramycin, cloxacillin, and fusidic acid.

**Results:**

The spectrophotometric MIC of manuka honey was 12% (w/v) for all tested isolates. MBC values ranged between 20% (w/v) and 28% (w/v) for all isolates, except *S. sciuri* . Combining 5% (w/v) manuka honey with tetracycline, chloramphenicol, or fusidic acid enhanced the antibacterial effect against *Staphylococcus* spp. (including methicillin‐resistant isolates). The antibacterial effect of ofloxacin against *S. aureus* and streptococci and of cloxacillin against *S. aureus* appeared slightly reduced when combined with 5% manuka honey. No consistent difference was observed when manuka honey was combined with gentamicin or tobramycin.

**Conclusions:**

Manuka honey has in vitro inhibitory and bactericidal activity against equine corneal surface pathogens including multi‐resistant isolates. Further studies are required to assess potential synergistic and antagonistic effects of manuka honey in combination with antibiotics.

## 1 Introduction

The development of antimicrobial resistance remains a concern in both human and veterinary medicine, presenting a challenging One Health issue. In ophthalmology, for instance, many bacterial pathogens associated with ulcerative corneal disease are resistant to commonly prescribed ophthalmic antibiotic agents [1, 2, 3, 4, 5, 6, 7]. *Streptococcus* spp., *Staphylococcus* spp., and *Pseudomonas aeruginosa* are the most commonly cultured bacterial isolates in equine ulcerative keratitis [5, 8, 9, 10, 11]. A high prevalence of methicillin‐resistant staphylococci as well as a frequent occurrence of multidrug‐resistant strains has been observed [5, 10, 12]. Furthermore, an association between acquired antibacterial resistance and prior topical antibacterial treatment has been reported [8, 9, 11]. The challenge of increasing antimicrobial resistance combined with the limited number of available topical antimicrobial agents emphasizes the importance of exploring alternative or adjunctive treatment options for infected corneal ulcerations in horses [13, 14].

Honey is well known for its antibacterial activity. However, with the advent of antibiotics, the clinical use of honey in conventional medicine has largely been neglected. Studies reporting the potent activity of honey against antibiotic‐resistant bacteria resulted in renewed interest in its application [15, 16]. In addition to its antibacterial properties, honey has antioxidant and anti‐inflammatory effects, which promote wound healing [17, 18]. The accelerated corneal wound healing in the presence of honey, as demonstrated in several in vitro and in vivo studies, further substantiates the potential of honey in treating corneal ulcerative disease [19, 20, 21, 22].

The antibacterial properties of honey are reportedly due to a combination of factors, including pH shock, osmotic activity, and the presence of hydrogen peroxide and non‐peroxide factors [18]. These non‐peroxide factors vary between different kinds of honey and consist of small molecules such as phenolics, flavonoids, and antibacterial peptides. Manuka honey is a monofloral honey derived from *Leptospermum scoparium* (Myrtaceae), an indigenous shrub species in New Zealand [23]. It is one of the major medical‐grade kinds of honey currently approved for clinical application. Methylglyoxal has been identified as the dominant antimicrobial component of manuka honey [24]. Due to the unique multiple antibacterial actions of manuka honey, microbial resistance has not yet been reported, even when sub‐inhibitory concentrations are used [25, 26]. This is in contrast to conventional antimicrobials, where sub‐inhibitory concentrations readily induce resistance [27, 28]. In addition, synergistic interactions between manuka honey and several antibiotics, for example, oxacillin, tetracycline, imipenem, mupirocin, and rifampicin, have been reported against multiple bacterial isolates including methicillin‐resistant *Staphylococcus aureus* (MRSA) [29, 30, 31, 32]. Interestingly, Jenkins and Cooper demonstrated that sub‐inhibitory concentrations of manuka honey could even restore susceptibility of MRSA to oxacillin [30]. These reports emphasize the potential of manuka honey as an antimicrobial adjuvant in combinational therapy.

Studies regarding the use of manuka honey in animals with ulcerative corneal disease are lacking. The objective of the current study was to investigate the antibacterial activity of manuka honey against resistant ocular pathogens and other clinically relevant bacterial strains frequently reported in infected ulcerative keratitis in horses. Additionally, the combined antibacterial effects of commonly prescribed topical antimicrobials and manuka honey were explored.

## 2 Materials and Methods

### 2.1 Bacterial Isolates

Four *S. aureus* (of which three were methicillin‐resistant (MRSA)), two methicillin‐resistant coagulase‐negative staphylococci (MRSCN), one *S. sciuri* and one *S. haemolyticus* , and two *Streptococcus equi* subspecies *zooepidemicus* isolates, were selected from the collection of the Veterinary Microbiological Diagnostic Centre (VMDC) of Utrecht University (Utrecht, The Netherlands). All isolates were stored at −80°C and originated from previously submitted clinical samples from horses with ulcerative keratitis. *Staphylococcus aureus* ATCC 25923 and *Streptococcus pneumoniae* ATCC 49619 were used for internal quality control [33]. Bacteria were grown aerobically on blood agar (BA) plates (Columbia Agar with Sheep Blood PLUS; ThermoFisher Scientific) for 18–22 h at 37°C under static conditions, after which colonies were checked for purity. Working cultures were stored at 4°C for a maximum of 4 weeks, after which new cultures were grown from frozen stock.

### 2.2 Inhibitors

Sterile preservative‐free 100% manuka honey (Manuka G Sterile, Kruuse) was used. Prior to each experiment, a stock solution of 40% weight/volume (w/v) was prepared by adding prewarmed (±37°C) manuka honey to sterile distilled water.

Antimicrobials were selected based on their availability as a veterinary registered topical formulation and/or their clinical applicability in equine ophthalmology. Both antimicrobial disks (Sensi‐Disks, Becton, Dickinson, and Company [BD]) and E‐strips (ETEST, BioMérieux) were used. Antimicrobials included chloramphenicol (30 μg disks, 0.016–256 μg E‐strips), tetracycline (30 μg disks, 0.016–256 μg E‐strips), gentamicin (10 μg disks, 0.016–256 μg E‐strips), ofloxacin (5 μg disks, 0.002–32 μg E‐strips), tobramycin (10 μg disks, 0.016–256 μg E‐strips), cloxacillin (10 μg disks), and fusidic acid (5 μg disks). For cloxacillin and fusidic acid, no E‐strips were available. Antimicrobial disks and *E*‐test strips were stored at 4°C and were removed from the refrigerator 1–2 h before use to allow them to equilibrate to room temperature.

### 2.3 Determination of Minimum Inhibitory Concentration and Minimum Bactericidal Concentration

The minimum inhibitory concentration (MIC) of manuka honey was determined for all isolates using the broth microdilution method following the Clinical and Laboratory Standards Institute (CLSI) guidelines with minor adjustments based on previously published research [33, 34, 35]. After overnight incubation on BA plates, colonies of the cultured bacterial isolates as described were suspended in glass vials containing 6 mL sterile 0.85% saline solution with a sterile cotton collection swab (EUROTUBO; Deltalab). The turbidity of each bacterial suspension was assessed with a DEN‐1 McFarland Densitometer (Grant Instruments) and adjusted to 0.5 McFarland (1–2 × 10^8^ CFU/mL). Hereafter, suspensions were further diluted to approximately 2 × 10^6^ CFU/mL in four times cation‐adjusted Mueller–Hinton broth (4xCAMHB; BD BBL, ThermoFisher Scientific) for use in the assay. *Streptococcus* spp. isolates were diluted in a separate 4xCAMHB with 10% lysed horse blood (LHB; ThermoFisher Scientific). For each experiment, 96‐well microtiter plates (Greiner Bio‐one, PS, f‐bottom; ThermoFisher Scientific) were used. The wells were filled with a total volume of 200 μL, made up of 50 μL inoculum (final concentration of approximately 5 × 10^5^ CFU/mL) and ranging volumes of nuclease‐free molecular grade water and 40% (w/v) manuka honey. The wells contained final concentrations of manuka honey ranging in 4% increments from 4% to 28% (w/v). For each assay, the following controls were included: a sterility control: wells containing broth only (without manuka honey and inoculum); a growth control: wells containing broth and inoculum (without manuka honey); and a color control: wells containing broth and manuka honey (without inoculum).

Immediately after inoculation, the optical density (OD) of all microtiter plate wells was determined at 600 nm (*t*_0_) using a spectrophotometer (FLUOstar Omega; BMG Labtech). Plates were covered with a sterile cover plate (Greiner Bio‐one, PS; ThermoFisher Scientific) and subsequently incubated for 20 h (±2 h) at 37°C in a static aerobic condition. After 20 h of incubation, visual MICs were determined, that is, the lowest concentration of manuka honey for which there was no visible bacterial growth (by the naked eye). The OD measurement was repeated (*t*_20_) directly after. OD data were used to determine the relative growth in the tested wells at each honey dilution. To correct for the turbidity of manuka honey and lysed horse blood in combination with MH broth, OD values from the corresponding dilutions of the color control wells were first subtracted from the OD values of the test wells. These color‐corrected *t*_0_ values were then subtracted from their corresponding *t*_20_ values for each well to generate net ODs as described in the formula below:

$$\begin{array}{c} \mathrm{OD}\;\mathrm{net}=\left({\mathrm{OD}}_{20}\;\text{test well}–{\mathrm{OD}}_{20}\;\text{color control}\right)\\ \;–\left({\mathrm{OD}}_{0}\;\text{test well}–{\mathrm{OD}}_{0}\;\text{color control}\right) \end{array}$$

Relative growth in the tested wells at each honey dilution was then determined by the following formula:

$$\text{Relative growth}=100^{*}\;\left[\mathrm{OD}\;\mathrm{net}/\left(\mathrm{OD}\;\text{growth control}–\mathrm{OD}\;\text{sterility control}\right)\right]$$

MIC_95_ was recorded as the lowest concentration of manuka honey yielding a relative growth of 5% or less. OD heatmaps were constructed using the relative OD values.

Aliquots of 10 μL from each well without visual bacterial growth were subcultured on BA plates and incubated for 20 h at 37°C. The lowest concentration of manuka honey that caused a 99.99% reduction in bacterial growth, as shown by the absence of growth or the appearance of fewer than 5 colonies, was considered the Minimum Bactericidal Concentration (MBC).

The experiments described above were performed on three separate days to provide experimental replicates.

### 2.4 Determination of Combined Antibiotic‐Honey Effects by Agar Diffusion Tests

Antimicrobial susceptibility tests using the disk diffusion method were performed according to the CLSI guidelines [33]. Mueller–Hinton agar (MHA; BD) was prepared in 9 and 15 cm round Petri dishes, with and without the addition of manuka honey. Prewarmed (±37°C) liquified 100% manuka honey was added to the cooled, yet unsolidified (±50°C) autoclaved MHA to achieve a 5% (w/v) concentration, after which the Petri dishes were filled. This 5% (w/v) manuka honey concentration was previously described as sub‐inhibitory under these conditions for *Staphylococcus* spp. [29, 31] and *Streptococcus* spp. [36], which was confirmed in our MIC determinations (see Section 3). After preparation, the plates were stored at 4°C until use. On the day of the experiment, the MHA and MHA + 5% (w/v) manuka honey plates were inoculated with a 0.5 McFarland suspension for each isolate, after which the antimicrobial disks and E‐strips were dispensed onto the surface of the inoculated agar plate of the 9 cm and 15 cm Petri dishes, respectively. The plates were incubated at 35°C for 20 h (±2 h) after which the inhibition zones were measured. For *Streptococcus* spp. MHA was supplemented with 5% (v/v) defibrinated sheep blood, and plates were incubated in 5% CO_2_. For cloxacillin and fusidic acid, no E‐strips were commercially available. All agar diffusion tests were repeated on three separate days to obtain reliable results.

### 2.5 Statistical Analysis

The mode of replicates was used to report the visual MIC and MBC values. Mean and standard deviations were calculated for the relative growth of all replicate spectrophotometric data. For the disk diffusion data, mean values were selected, and for the *E*‐test data, median values were selected. For the disk diffusion and *E*‐test data, bar plots were created in R Statistical Software (version 4.4.1.2024). The antibacterial effect of manuka honey was classified as “additive” when a larger diameter for the disk diffusion and/or a lower MIC for the *E*‐test was found compared to the diameter of the control (without manuka honey) on the same day; results were classified as “reduced” when a smaller diameter for the disk diffusion and/or a higher MIC for the *E*‐test was found, and combinations that did not show an effect were classified as “indifferent”. Data from isolates were then grouped by bacterial species: *S. aureus* (5 isolates), MRSCN (2 isolates), and streptococci (3 isolates). A binomial test was performed in R (binom.test function) to assess the proportion of additive effects relative to the total of additive and reduced effects; indifferent effects were not taken into account. Under the null hypothesis that manuka honey has no effect, additive and reduced effects were expected to occur equally often (50:50). Consequently, the test assessed whether the observed proportion of additive effects deviated significantly from this expectation. This test was conducted separately for each antibiotic and bacterial species combination for each assay type (disk diffusion and E‐test), with statistical significance set at *p* < 0.05. A Benjamini–Hochberg correction was applied to all combined results with a false discovery rate (FDR) of 10%.

## 3 Results

### 3.1 Antimicrobial Activity of Manuka Honey

**FIGURE 1:**
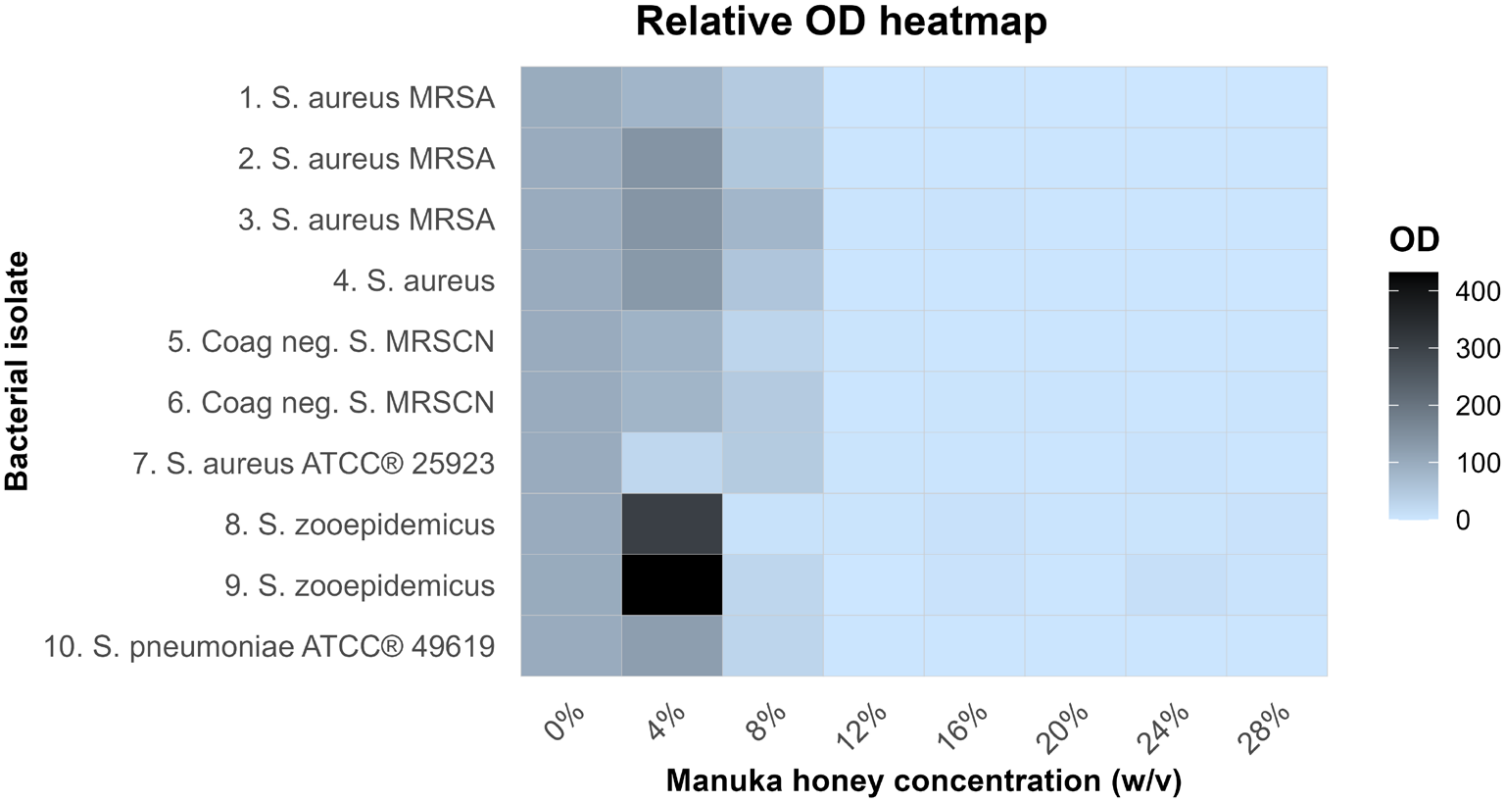
Relative optical density (OD) of eight bacterial strains isolated from infected corneal ulcerations in horses and two reference bacterial strains in increasing concentrations of manuka honey. Coag negative, coagulase‐negative; MRSA, methicillin‐resistant *Staphylococcus aureus* ; MRSCN, methicillin‐resistant coagulase‐negative *Staphylococcus*.

The visual MIC, spectrophotometric MIC_95_, and MBC values are presented in Table 1. Manuka honey exhibited an MIC_95_ of 12% (w/v) for all isolates. The lowest concentration of manuka honey (4% w/v) appeared to enhance the growth of both S. *zooepidemicus* isolates (430%) and, to a lesser extent, of three *S. aureus* isolates (up to 140%) as illustrated in Figure 1. For all but one bacterial isolate, the MBC was established to be between 20% and 28% (w/v); for the methicillin‐resistant *S. sciuri* isolate, no 99.9% reduction in bacterial growth was reached at the highest concentration (28%) used. The reference strain *S. pneumoniae* ATCC 49619 exhibited the lowest MBC, recorded at 8% (w/v).

**TABLE 1:** Visual MIC, spectrophotometric MIC_95_ and MBC (% w/v) of manuka honey for eight bacterial strains isolated from infected corneal ulcerations in horses and two reference bacterial strains.

Microorganism	MIC (%)	MIC_95_ (%)	MBC
1. *S. aureus* MRSA	12	12	24
2. *S. aureus* MRSA	12	12	24
4. *S. aureus* MRSA	12	12	24
3. *S. aureus*	12	12	28
5. MRSCN	12	12	> 28
6. MRSCN	12	12	20
7. *S. aureus* ATCC 25 923	12	12	24
8. S. *zooepidemicus*	8	12	20
9. S. *zooepidemicus*	8	12	24
10. *S. pneumoniae* ATCC 49 619	8	12	8

Abbreviations: MRSA, methicillin‐resistant *Staphylococcus aureus* ; MRSCN, methicillin‐resistant coagulase‐negative *Staphylococcus*.

### 3.2 Antibiotic Susceptibility in the Absence and Presence of Manuka Honey

**FIGURE 2:**
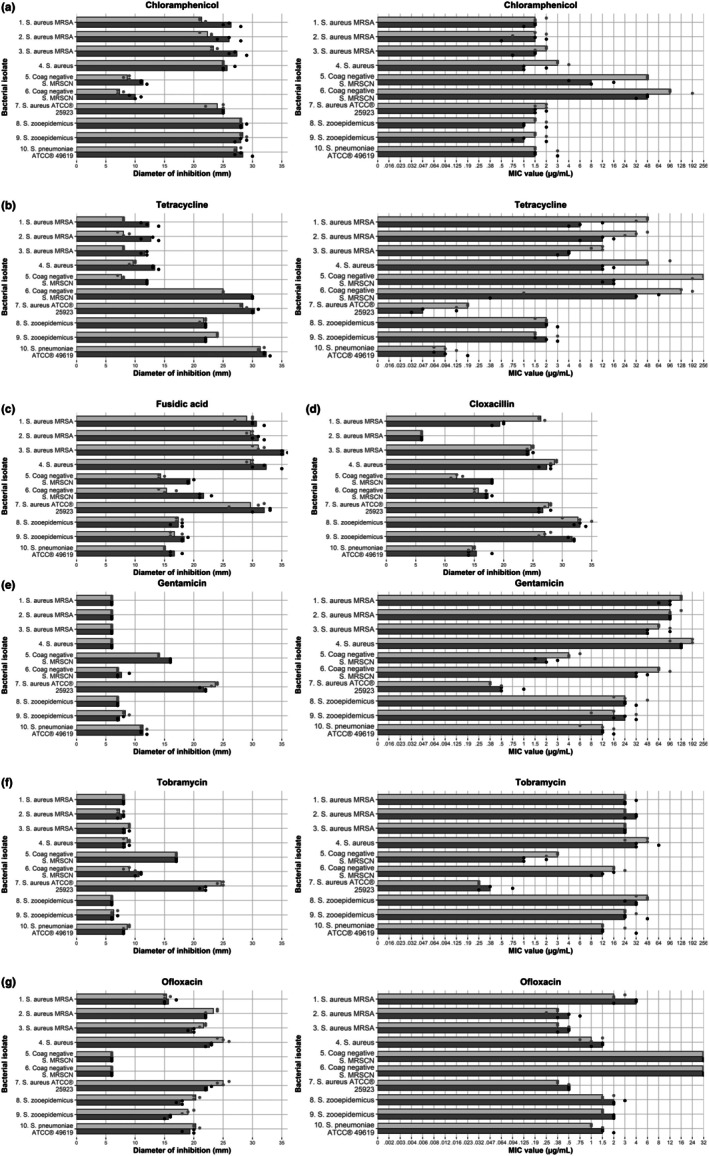
Disk diffusion (left) and E‐test (right) results of the tested antibiotics without (light gray) and with (dark gray) 5% (w/v) manuka honey for eight bacterial strains isolated from infected corneal ulcerations in horses and one reference bacterial strain. Dots represent individual measurements. For the disk diffusion test, a higher diameter represents increased inhibition, for the E‐test, a lower MIC represents increased inhibition. (a) Chloramphenicol. (b) Tetracyclin. (c) Fusidic acid (disk diffusion). (d) Cloxacillin (disk diffusion). (e) Gentamicin. (f) Tobramycin. (g) Ofloxaxin.

Figure 2 illustrates the results of the disk diffusion assay and E‐tests, both in the absence and presence of a sub‐inhibitory concentration (5% w/v) of manuka honey, for all antibiotics tested.

For both *S. aureus* and MRSCN, the binomial test showed that the proportion of results with an additive effect of 5% manuka honey differed significantly from the expected 50% when combined with chloramphenicol, tetracycline, and fusidic acid (Tables 2 and 3). A similar significant additive inhibitory effect of 5% manuka honey was observed in MRSCN isolates when combined with cloxacillin and gentamicin (E‐test only).

**TABLE 2:** Number of observations per bacterial species group and number, percentage, *p*‐value and conclusion of additive, reduced and indifferent effect of 5% manuka honey combined with different antibiotics using disk diffusion test.

Antibiotic	Bacteria	*n*	Additive, *n* (%)	Reduced, *n* (%)	Indifference, *n* (%)	*p*	Significant effect
Chloramphenicol	*S. aureus*	15	11 (73.3)	0 (0.0)	4 (26.7)	0.001	Additive
Chloramphenicol	MRSCN	6	6 (100.0)	0 (0.0)	0 (0.0)	0.031	Additive
Chloramphenicol	Streptococci	9	3 (33.3)	1 (11.1)	5 (55.6)	0.625	
Tetracycline	*S. aureus*	15	15 (100.0)	0 (0.0)	0 (0.0)	< 0.001	Additive
Tetracycline	MRSCN	6	6 (100.0)	0 (0.0)	0 (0.0)	0.031	Additive
Tetracycline	Streptococci	9	3 (33.3)	3 (33.3)	3 (33.3)	1.000	
Fusidic acid	*S. aureus*	15	13 (85.6)	0 (0.0)	2 (13.3)	< 0.001	Additive
Fusidic acid	MRSCN	6	6 (100.0)	0 (0.0)	0 (0.0)	0.031	Additive
Fusidic acid	Streptococci	9	7 (77.8)	1 (11.1)	1 (11.1)	0.070	
Cloxacillin	*S. aureus*	15	0 (0.0)	8 (53.3)	7 (46.7)	0.008	Reduced
Cloxacillin	MRSCN	6	6 (100.0)	0 (0.0)	0 (0.0)	0.031	Additive
Cloxacillin	Streptococci	9	5 (55.6)	3 (33.3)	1 (11.1)	0.727	
Gentamicin	*S. aureus*	15	0 (0.0)	3 (20.0)	12 (80.0)	0.250	
Gentamicin	MRSCN	6	4 (66.7)	0 (0.0)	2 (33.3)	0.125	
Gentamicin	Streptococci	9	0 (0.0)	2 (22.2)	7 (77.8)	0.500	
Tobramycin	*S. aureus*	15	1 (6.7)	6 (40.0)	8 (53.3)	0.125	
Tobramycin	MRSCN	6	2 (33.3)	0 (0.0)	4 (66.7)	0.500	
Tobramycin	Streptococci	9	0 (0.0)	2 (22.2)	7 (77.8)	0.500	
Ofloxacin	*S. aureus*	15	1 (6.7)	12 (80.0)	2 (13.3)	0.003	Reduced
Ofloxacin	MRSCN	6	0 (0.0)	0 (0.0)	6 (100.0)	NA	
Ofloxacin	Streptococci	9	0 (0.0)	8 (88.9)	1 (11.1)	0.008	Reduced

*Note:* Tested bacteria were classified in groups ( *S. aureus* , coagulase‐negative Staphycolocci (MRSCN) and *Streptococci*). *p* values were determined with a binomial test with a Benjamini‐Hochberg correction with a fractional discrimination rate of 10%. A significant effect was noted with a *p* < 0.05.

**TABLE 3:** Number of observations per bacterial species group and number, percentage, *p*‐value and conclusion of additive, reduced and indifferent effect of 5% manuka honey combined with different antibiotics using E‐test.

Antibiotic	Bacteria	*n*	Additive, *n* (%)	Reduced, *n* (%)	Indifference, *n* (%)	*p*	Significant effect
Chloramphenicol	*S. aureus*	15	11 (73.3)	1 (6.7)	3 (20.0)	0.006	Additive
Chloramphenicol	MRSCN	6	6 (100.0)	0 (0.0)	0 (0.0)	0.031	Additive
Chloramphenicol	Streptococci	9	4 (44.4)	0 (0.0)	5 (55.6)	0.125	
Tetracycline	*S. aureus*	15	15 (100.0)	0 (0.0)	0 (0.0)	< 0.001	Additive
Tetracycline	MRSCN	6	6 (100.0)	0 (0.0)	0 (0.0)	0.031	Additive
Tetracycline	Streptococci	9	0 (0.0)	4 (44.4)	5 (55.6)	0.125	
Gentamicin	*S. aureus*	15	8 (53.3)	4 (26.7)	3 (20.0)	0.388	
Gentamicin	MRSCN	6	6 (100.0)	0 (0.0)	0 (0.0)	0.031	Additive
Gentamicin	Streptococci	9	1 (11.1)	4 (44.4)	4 (44.4)	0.375	
Tobramycin	*S. aureus*	15	2 (13.3)	6 (40.0)	7 (46.7)	0.289	
Tobramycin	MRSCN	6	5 (83.3)	0 (0.0)	1 (16.7)	0.063	
Tobramycin	Streptococci	9	3 (33.3)	2 (22.2)	4 (44.4)	1000	
Ofloxacin	*S. aureus*	15	1 (6.7)	10 (66.7)	4 (26.7)	0.012	Reduced
Ofloxacin	MRSCN	6	0 (0.0)	0 (0.0)	6 (100.0)	NA	
Ofloxacin	Streptococci	9	0 (0.0)	9 (100.0)	0 (0.0)	0.004	Reduced

*Note:* Tested bacteria were classified in groups ( *S. aureus* , coagulase‐negative Staphycolocci [MRSCN] and Streptococci). *p* values were determined with a binomial test with a Benjamini–Hochberg correction with a fractional discrimination rate of 10%. A significant effect was noted with a *p* < 0.05.

For *S. aureus* and streptococci, the binomial test showed that the proportion of results with a reduced effect of 5% manuka honey differed significantly from the expected 50% for ofloxacin. For *S. aureus* , a significantly reduced inhibitory effect of 5% manuka honey was observed when combined with cloxacillin.

No significant additive or reduced effect of adding 5% manuka honey was observed for the other antimicrobials tested.

## 4 Discussion

In this in vitro study, manuka honey effectively inhibited the growth of bacteria commonly found in equine ulcerative keratitis, regardless of their antimicrobial resistance profile. Additionally, manuka honey was shown to be bactericidal in concentrations ranging from 8% to 28% (w/v) for all tested isolates except one MRSCN. The determined MIC of 12% (w/v) for all staphylococci (including methicillin‐resistant isolates) in the present study is consistent with formerly reported MICs ranging from 4% to 12.5% (w/v) [26, 30, 32, 37, 38, 39]. The slightly lower MIC for *S. equi* ssp. *zooepidemicus* (8% [w/v]) also falls within the previously observed MIC range of 4%–10% (w/v) [39].

Due to the turbidity and slightly yellowish color of manuka honey, visual inspection of bacterial growth in the 96‐well microtiter plate proved challenging to interpret. Spectrophotometric evaluation was considered a more objective and reproducible method for determining the MIC. This approach has been previously utilized to quantify antibacterial activity, including that of honey [40, 41]. Spectrophotometry was particularly useful in the wells containing *Streptococcus* spp., where the presence of lysed horse blood hampered the visual MIC interpretation even more [34]. Using control wells to correct OD values for color and turbidity caused by manuka honey and lysed horse blood allowed for the quantification of relative growth and proved to be a more sensitive method compared to visual inspection.

To define whether an antibacterial agent is bactericidal or bacteriostatic in vitro, the MBC:MIC ratio can be used. If the MBC:MIC ratio is ≤ 4, the effect is considered bactericidal, and if the MBC:MIC ratio is > 4, the effect is defined as bacteriostatic. In the current study, although MBC values were considerably higher (≥ 20% [w/v]) than MIC values (12% [w/v]), the MBC:MIC ratios for all but one bacterial isolate were ≤ 4, and therefore, the effect of manuka honey was considered to be bactericidal. One MRSCN was able to grow in manuka honey concentrations up to 28% (w/v). However, its growth was stunted in the 24% and 28% (w/v) concentrations: upon subculture on BA, only a few (< 10) malformed colonies grew. Higher concentrations of manuka honey were not tested, so an MBC could not be determined for this isolate.

Although manuka honey demonstrated promising antibacterial activity at concentrations of 12% (w/v) or more, spectrophotometric data from the present study revealed that a 4% (w/v) concentration of manuka honey appeared to enhance bacterial growth, particularly in *Streptococcus* spp. and to a lesser extent in *S. aureus* . This finding aligns with a study on the antibacterial effects of several honey types against impetigo‐associated pathogens, where low concentrations (< 16% [w/v]) of Wandoo and multifloral honey enhanced the growth of *S. aureus* and MRSCN [38]. A similar effect, though to a lesser extent, was observed at lower concentrations (< 4% [w/v]) of manuka honey in another study [42]. The enhancement of bacterial growth in the presence of low concentrations of manuka honey warrants further exploration in a clinical setting, as topical administration to the eye will ultimately lead to dilution of the antimicrobial compound due to tear film washout. The in vitro determination of the antibacterial effect has its limitations and can be affected by multiple variables. Time‐kill assays can provide additional information on whether bacterial killing is concentration‐ or time‐dependent. Still, clinical studies are needed to explore the pharmacodynamic properties of manuka honey in vivo.

The observed additive antibacterial effect of a subinhibitory concentration of manuka honey when combined with chloramphenicol, tetracycline or fusidic acid against staphylococci justifies further investigation into potential clinical applications, as staphylococci are commonly found in ocular infections [7, 8, 10, 12, 43, 44, 45]. Similar synergistic combined effects of manuka honey and antibiotics against *S. aureus* , including MRSA, have been reported earlier for several antibiotics, including oxacillin, rifampicin, tetracycline, imipenem, clindamycin, and mupirocin [29, 32, 46]. The present study did not observe any additive effect when manuka honey was combined with tobramycin and only an additive effect for MRSCN when combined with gentamicin. Two studies investigating potential synergistic interactions between antimicrobials and 5% (w/v) manuka honey, using agar diffusion and/or chequerboard assays, found no additive effect when honey was combined with tobramycin or gentamicin [29, 32]. Contradicting results were found in a different study using chequerboard microdilution assays, where additive effects of manuka honey combined with gentamicin against *S. aureus* were observed at concentrations of 6%–10% [47]. The finding of re‐sensitization of MRSA strains to oxacillin by downregulation of the *mecR1* gene was not confirmed in our study where we used another isoxazolyl penicillin, cloxacillin, with and without manuka honey. In this study, a significant additive effect of 5% manuka honey in MRSCN isolates and a reduced effect in *S. aureus* isolates (including three methicillin‐resistant isolates) when combined with cloxacillin was observed. Tests to investigate the *mecR1* pathway in these isolates could give more insight into the mechanism behind these effects. Surprisingly, for ofloxacin—a commonly prescribed ocular agent, especially in countries with less restrictive guidelines for antibiotic use, and the only fluoroquinolone antimicrobial used in this study—a reduced effect was observed when combined with manuka honey. This reduced effect warrants further investigation into potential antagonistic properties of (manuka) honey, as, to the authors' knowledge, this has not yet been reported.

In this study, no apparent increase or reduction in MIC values or inhibition zone diameter was measured with the addition of 5% (w/v) manuka honey to the selected antibiotics against *Streptococcus* spp., with the exception of ofloxacin, which showed a significantly reduced antibacterial effect when combined with manuka honey. In hindsight, the 5% concentration of manuka honey may not have been truly subinhibitory, as spectrophotometric data revealed up to 430% growth increase of streptococci in the presence of 4% manuka honey. Repeating the disk diffusion and *E*‐test experiments with higher concentrations of manuka honey could provide more insight into the potential combinatorial effects of antibacterial drugs against streptococci.

Variations in the results of different studies can be attributed to several factors, including bacterial strain variability, differences in antimicrobial combinations and concentrations, types and concentrations of honey used, testing environment, and the testing methods employed. These variations hamper the comparability of outcomes.

Drug penetration into the cornea is a complex process, influenced by various factors such as the physicochemical properties of the drug, the corneal structure, and the presence of the tear film, all of which can impact the efficacy of topical treatments. The corneal epithelium presents a significant barrier due to the presence of tight junctions and because of its lipophilic nature, which limits the penetration of hydrophilic drugs. However, when the epithelium is disrupted, hydrophilic compounds can be more effectively delivered into the cornea. Because of its high sugar content, honey is very hydrophilic, which potentiates its corneal penetration in ulcerative keratitis. In humans, topical application of a 98% and 16% solution (w/w) of manuka honey has been reported to cause transient ocular stinging and redness upon instillation, lasting up to 5 min. However, these side effects were minimized after repeated application and the majority of patients reported good compliance with the treatment [48, 49]. Additionally, studies using either 20% honey eyedrops for the treatment of dry eye syndrome, or 100% honey for corneal edema in humans reported no adverse reactions other than a temporary local stinging sensation in some patients [50, 51]. Although several ophthalmic formulations containing honey are currently on the market, the authors are not aware of any clinical applications in animals, including horses. Future studies on the application of honey eyedrops and/or eye gel in equine eyes would be valuable for assessing its clinical applicability and potential side effects in this species.

Infected corneal ulcerations in horses often require a multimodal treatment approach. Therefore, in addition to providing MIC and MBC values of manuka honey against equine corneal surface pathogens, this study aimed to investigate the combined antibacterial effect of manuka honey and several antibiotics. It provides only preliminary insights into this combined antibacterial effect, since the small sample size, the limited number of bacterial isolates and antibiotics tested, and the use of only one concentration of manuka honey hamper firm quantitative conclusions. Additional in vitro tests, such as chequerboard testing, would enable the calculation of the Fractional Inhibitory Concentration Index values, providing a more quantitative assessment of the combined antibacterial effects of manuka honey and antimicrobials, including the determination of potential antagonistic or synergistic effects. The findings of the current study may serve as a basis for future research.

## 5 Conclusion

Manuka honey at concentrations of 12% (w/v) or higher has in vitro antibacterial activity against bacteria, including methicillin‐resistant strains, that are commonly associated with infected corneal ulcerations in horses. This suggests that manuka honey could potentially be a valuable complement or alternative to existing antibacterial treatment for infected corneal ulcerations in horses. At the sub‐inhibitory concentration of 5% (w/v), manuka honey enhanced the antibacterial effect of tetracycline, chloramphenicol and fusidic acid against *Staphylococcus* spp. (including methicillin‐resistant isolates), whereas it appeared to slightly reduce the antibacterial effect of ofloxacin against both staphylococci and streptococci. These preliminary findings warrant future studies into the combined synergistic or antagonistic effects of manuka honey and topical antibiotics. Further in vitro and subsequent in vivo testing is needed to fully assess the applicability of manuka honey as a treatment for infected corneal ulcerations in horses.

## Disclosure

Artificial Intelligence Statement: The authors have not used AI to generate any part of the manuscript.

## Ethics Statement

The authors have nothing to report.

## Conflicts of Interest

The authors declare no conflicts of interest.

## Data Availability

The data that support the findings of this study are available from the corresponding author upon reasonable request.
